# Paediatrics hospitalization profile in England: A longitudinal ecological study

**DOI:** 10.1097/MD.0000000000043113

**Published:** 2025-07-04

**Authors:** Asaleh El-Qasem, Esra’ O. Taybeh, Hassan Alwafi, Adnan Taybeh, Alaa A. Alsharif, Ahmed M. Al Rajeh, Jaber S. Alqahtani, Abdulelah M. Aldhahir, Abdullah A. Alqarni, Anan S. Jarab, Yosra J. Alhartani, Abdallah Y. Naser

**Affiliations:** a Faculty of Pharmacy, University of Jordan, Amman, Jordan; b Department of Applied Pharmaceutical Sciences and Clinical Pharmacy, Faculty of Pharmacy, Isra University, Amman, Jordan; c Department of Clinical Pharmacology and Toxicology, Faculty of Medicine, Umm Al-Qura University, Makkah, Saudi Arabia; d Al-Bashir Hospital, Ministry of Health, Amman, Jordan; e Department of Pharmacy Practice, College of Pharmacy, Princess Nourah bint Abdulrahman University, Riyadh, Saudi Arabia; f Department of Respiratory Care, College of Applied Medical Sciences, King Faisal University, Al-Ahsa, Saudi Arabia; g Department of Respiratory Care, Prince Sultan Military College of Health Sciences, Dammam, Saudi Arabia; h Respiratory Therapy Department, Faculty of Applied Medical Sciences, Jazan University, Jazan, Saudi Arabia; i Department of Respiratory Therapy, Faculty of Medical Rehabilitation Sciences, King Abdulaziz University, Jeddah, Saudi Arabia; j Department of Clinical Pharmacy, Faculty of Pharmacy, Jordan University of Science and Technology, Irbid, Jordan.

**Keywords:** admission, children, England, hospitalization, pediatrics

## Abstract

Children constitute a large proportion of any population. For the appropriate allocation of resources to promote pediatric health, it is necessary to comprehend the patterns of healthcare usage among pediatric patients. This research aimed to determine the hospitalization among England’s pediatric population. This ecological study examined the hospitalization profile of the under-15-year-old pediatric population in England. For this investigation, data were extracted from the Hospital Episode Statistics database in England. Using the Pearson chi-square test for independence, the variance in hospitalization rates between 2012 and 2020 was determined. In England, hospital admission episodes among pediatrics totaled 19,905,606 during the duration of the study, with a mean of 2488,201 each year. The overall annual number of hospital admission among pediatrics for different causes decreased by 0.3% from 2468,570 in 2012 to 2460,755 in 2020, representing a decrease in hospital admission rate among pediatrics of 4.0% (from 19,235.82 [95% confidence interval: 19,214.25–19,257.38] in 2012 to 18,459.79 [95% confidence interval: 18,438.96–18,480.61] in 2020 per 100,000 persons, *P* ≥ .05). Amongst pediatric patients, 3 of the most common causes of admission were factors influencing health status and contact with health services, certain conditions originating in the perinatal period, and diseases of the respiratory system, accounting for 11.8% and 11.2%, respectively. Most pediatric admissions were more common in the 0 to 4 years age range (59.4%). This study shows that pediatric hospital admissions in England decreased, while nervous system, blood, and immunological problems increased, notably in the 0 to 4 age group. Healthcare providers should prioritize preventative care and early intervention for vulnerable children and increasing conditions. To improve pediatric health outcomes and healthcare methods, future studies should examine these increases’ origins, socio-economic determinants, and preventive interventions.

## 1. Introduction

In 2019, the global burden of communicable diseases among children and adolescents was 3.0 million fatalities, which accounted for 57.3% of the total burden across all ages. There was a significant burden of communicable disease morbidity in high- and high-middle-income countries, in addition to the predominant disease burden and mortality in low-income countries.^[1]^ The global communicable disease burden in children and adolescents was 59·8% due to the primary 3 causes: enteric infections, lower-respiratory-tract infections, and malaria.^[1]^ Pediatric mortality and morbidity trends provide indicative data regarding the health state. These indicators assist in directing resources and establishing objectives for healthcare sectors that require handling with the highest priority by emphasizing the primary influencing factors; that contribute to the rise in death and morbidity.^[2]^

Effectual health systems are based on primary care; nevertheless, hospital services and primary care are experiencing increasing strain worldwide.^[3]^ In terms of expressing the part of the health system that consumes the most resources, hospital morbidity, which reflects the considerably severe category of disorders and individuals who have admission to hospitals, has a specific significance.^[4]^ Additionally, according to some experts, data on hospital admissions rates and reasons is an indirect pointer to determining the accuracy of outpatient consultations and a critical sign of the quality of the services provided.^[5]^

In the UK, since the late 1990s, there has been a sharp rise in the number of children using hospital services.^[6–8,9]^ According to a previous study by Naser, during the past 2 decades, 11.7% of all hospital admissions in England and Wales were for patients aged <15 years.^[4]^ Accordingly, this study aims to describe the characteristics of hospital admissions for pediatric patients in England.

## 2. Method

### 2.1. Study design

This was a retrospective ecological study using population-level data on pediatric patients that were accessible to the public. We utilized data from the Hospital Episode Statistics database in England between April 1, 2012, and April 1, 2020, for the present investigation.^[10]^ Prior to 2012, data were published for patients aged under 15 altogether, not stratified for specific pediatric age groups (0–4 yr, 5–9 yr, and 10–14 yr). The Hospital Episode Statistics database contains information on pediatric patients admitted to hospitals for any medical reason.

### 2.2. Data extraction

We used the Tenth Revision of the International Statistical Classification of Illnesses and Related Health Problems (ICD), ICD-10 5th Edition, applied by the National Health Service in identifying diseases and other health problems. We used the database available at the Office for National Statistics of mid-year population data from 2012 to 2020 to assess the trend of annual hospitalization for pediatric hospital admissions, A00-Z99, every year. This was further queried with the database now available at the Office for National Statistics from the mid-year population data for the years 2012 to 2020 to ascertain the trend of annual hospitalization.

### 2.3. Statistical analysis

Descriptive statistics were employed to describe the categorical data in terms of frequency and percentage. Hospital admission rate was calculated by dividing the number of pediatric patient admissions as the nominator by the mid-year pediatric population as the denominator, along with 95% confidence intervals (CIs). Similar methods were applied to estimate the admission rates, stratified by gender and sub-age. We then compared the hospital admission rates between 2012 to 2020 by using a chi-squared test. All analyses were performed in SPSS, version 27, from IBM Corp., Armonk, NY.

## 3. Results

In England, hospital admission episodes among pediatrics totaled 19,905,606 during the duration of the study, with a mean of 2488,201 each year. The overall annual number of hospital admission among pediatrics for different causes decreased by 0.3% from 2468,570 in 2012 to 2460,755 in 2020, representing a decrease in hospital admission rate among pediatrics of 4.0% (from 19,235.82 [95%CI: 19,214.25–19,257.38] in 2012 to 18,459.79 [95%CI: 18,438.96–18,480.61] in 2020 per 100,000 persons, *P* ≥ .05).

Amongst pediatric patients, 3 of the most common causes of admission were factors influencing health status and contact with health services, certain conditions originating in the perinatal period, and diseases of the respiratory system, accounting for 21.1 percent, 11.8 percent, and 11.2 percent, respectively (Table 1).

**Table 1:** Percentage hospital admission among pediatric from total number of admissions.

ICD code	Indication	Percentage
P00–P96	“Certain conditions originating in the perinatal period”	11.8%
J00–J99	“Diseases of the respiratory system”	11.2%
R00–R99	“Symptoms, signs and abnormal clinical and laboratory findings”	9.0%
K00–K93	“Diseases of the digestive system”	7.5%
A00–B99	“Certain infectious and parasitic diseases”	6.5%
Q00–Q99	“Congenital malformations, deformations and chromosomal abnormalities”	3.8%
N00–N99	“Diseases of the genitourinary system”	3.1%
C00–D48	“Neoplasms”	2.9%
M00–M99	“Diseases of the musculoskeletal system and connective tissue”	2.5%
O00–O99	“Pregnancy, childbirth and the puerperium”	2.5%
G00–G99	“Diseases of the nervous system”	1.9%
H60–H95	“Diseases of the ear and mastoid process”	1.6%
L00–L99	“Diseases of the skin and subcutaneous tissue”	1.6%
D50–D89	“Diseases of the blood and blood-forming organs and certain disorders involving the immune mechanism”	1.5%
E00–E90	“Endocrine, nutritional and metabolic diseases”	1.5%
H00–H59	“Diseases of the eye and adnexa”	1.0%
F00–F99	“Mental and behavioral disorders”	0.7%
I00–I99	“Diseases of the circulatory system”	0.6%
S00–T98	“Injury, poisoning and certain other consequences of external causes”	7.7%
Z00–Z99	“Factors influencing health status and contact with health services”	21.1%

ICD = International Statistical Classification of Illnesses and Related Health Problems.

**Figure 1.**
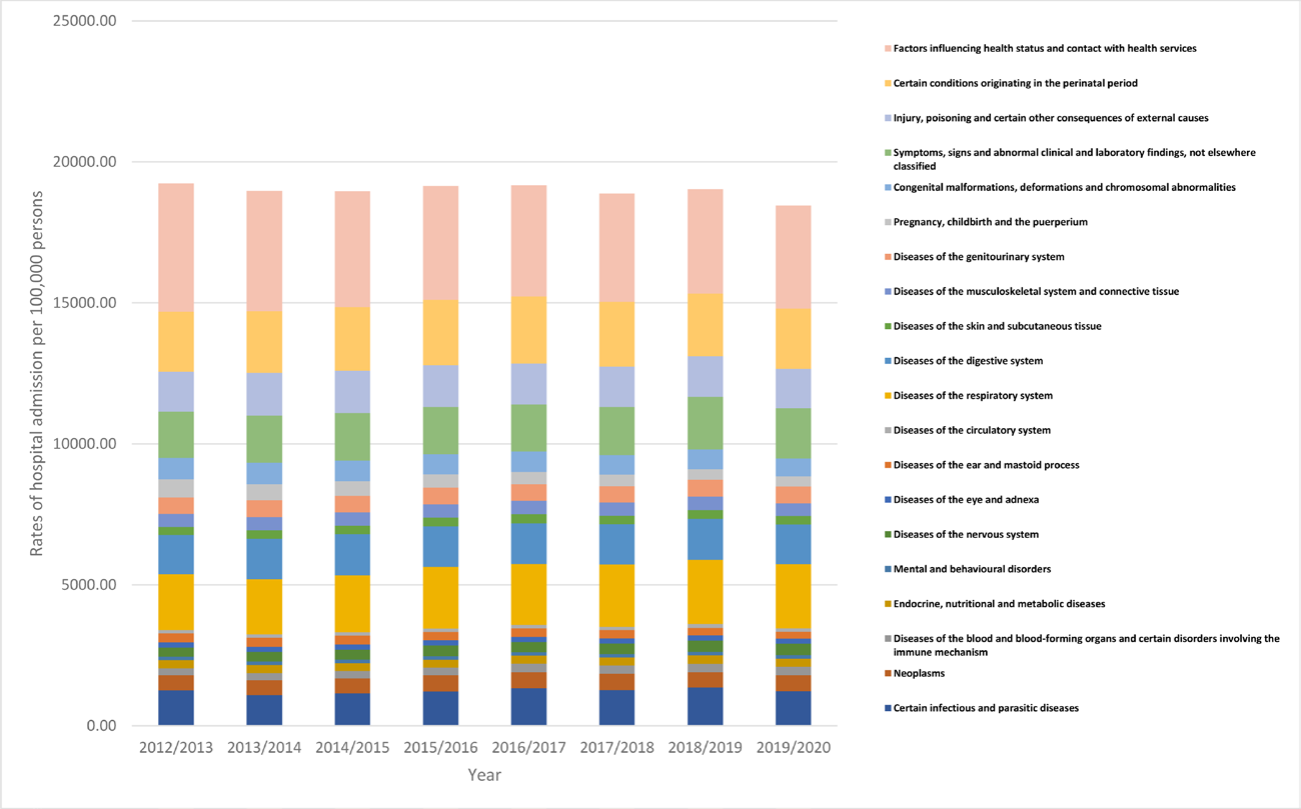
Admission rate stratified by type.

During the period under study, the admission rate of hospital pediatrics with diseases of the nervous system, diseases of the blood and blood-forming organs, and certain disorders involving the immune mechanism, diseases of the respiratory system, and diseases of the circulatory system increased by 25.3%, 16.4%, 14.4%, and 13.0%, respectively. Admissions to the hospital by pediatrics for symptoms, signs, and abnormal clinical and laboratory findings, diseases of the skin and subcutaneous tissue, neoplasms, mental and behavioral disorders, diseases of the genitourinary system, and diseases of the digestive system increased by 9.0%, 8.3%, 6.5%, 5.4%, 2.7%, and 0.9%, respectively. However, hospital admission rates for pediatric pregnancy, childbirth, and the puerperium, diseases of the ear and mastoid process, factors influencing health status and contact with health services, congenital malformations, deformations and chromosomal abnormalities, diseases of the musculoskeletal system and connective tissue, diseases of the eye and adnexa, certain infectious and parasitic diseases, injury, poisoning and certain other consequences of external causes, endocrine, nutritional and metabolic diseases, and certain conditions originating in the perinatal period were reduced by 46.0%, 24.6%, 19.3%, 15.4%, 2.8%, 2.6%, 2.3%, 1.5%, 0.2%, and 0.1%, respectively (Fig. 1, Table 2).

**Table 2:** Change in admission rate among paediatrics from 2012–2020 in England.

Conditions	Rate of conditions in 2012 per 100,000 persons (95% CI)	Rate of conditions in 2020 per 100,000 persons (95% CI)	Percentage change
“Diseases of the nervous system”	327.71 (324.59–330.84)	410.50 (407.07–413.93)	25.3%
“Diseases of the blood and blood-forming organs and certain disorders involving the immune mechanism”	254.75 (251.99–257.50)	296.61 (293.69–299.53)	16.4%
“Diseases of the respiratory system”	1980.87 (1973.25–1988.49)	2267.03 (2259.04–2275.02)	14.4%
“Diseases of the circulatory system”	109.54 (107.73–111.35)	123.78 (121.89–125.67)	13.0%
“Symptoms, signs and abnormal clinical and laboratory findings”	1636.56 (1629.62–1643.50)	1784.13 (1777.02–1791.24)	9.0%
“Diseases of the skin and subcutaneous tissue”	291.87 (288.92–294.82)	316.15 (313.14–319.16)	8.3%
“Neoplasms”	525.20 (521.25–529.16)	559.56 (555.55–563.56)	6.5%
“Mental and behavioral disorders”	121.38 (119.48–123.29)	127.88 (125.96–129.80)	5.4%
“Diseases of the genitourinary system”	580.62 (576.46–584.78)	596.44 (592.31–600.58)	2.7%
“Diseases of the digestive system”	1392.60 (1386.19–1399.01)	1405.53 (1399.21–1411.85)	0.9%
“Certain conditions originating in the perinatal period”	2134.91 (2127.00–2142.82)	2132.60 (2124.84–2140.35)	−0.1%
“Endocrine, nutritional and metabolic diseases”	286.99 (284.06–289.92)	286.44 (283.57–289.31)	−0.2%
“Injury, poisoning and certain other consequences of external causes”	1419.14 (1412.67–1425.61)	1397.87 (1391.57–1404.17)	−1.5%
“Certain infectious and parasitic diseases”	1260.99 (1254.88–1267.09)	1232.19 (1226.27–1238.11)	−2.3%
“Diseases of the eye and adnexa”	183.31 (180.97–185.65)	178.52 (176.26–180.79)	−2.6%
“Diseases of the musculoskeletal system and connective tissue”	458.03 (454.34–461.73)	445.12 (441.55–448.69)	−2.8%
“Congenital malformations, deformations and chromosomal abnormalities”	748.01 (743.30–752.73)	632.67 (628.41–636.93)	−15.4%
“Factors influencing health status and contact with health services”	4543.40 (4532.00–4554.79)	3667.68 (3657.59–3677.77)	−19.3%
“Diseases of the ear and mastoid process”	325.58 (322.46–328.69)	245.42 (242.76–248.07)	−24.6%
“Pregnancy, childbirth and the puerperium”	654.36 (649.95–658.77)	353.67 (350.49–356.86)	−46.0%

CI = confidence interval.

**Figure 2.**
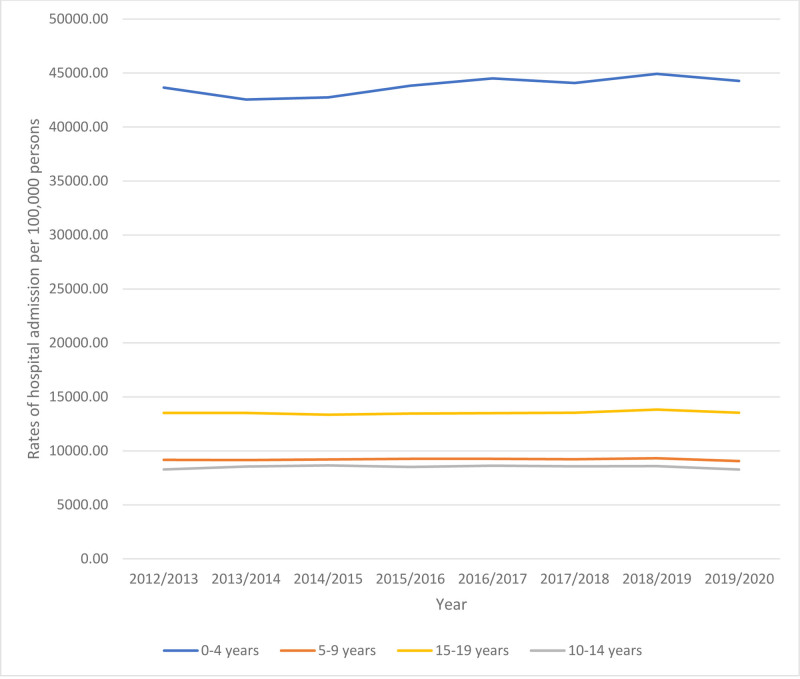
Overall admission rate stratified by age group.

Regarding differences in the age group hospital admission among pediatrics, the age group 0 to 4 years accounted for 59.4% of the total number of hospital admissions among pediatrics, followed by the age group 15 to 19 years with 17.2%, the age group 5 to 9 years with 12.6%, and then the age group 10 to 14 years with 10.8%. Admission rate among pediatrics aged 0 to 4 years increased by 1.4% (from 43,647.46 [95%CI: 43,594.85–43,700.07] in 2012 to 44,276.08 [95%CI: 44,221.98–44,330.17] in 2020 per 100,000 persons). Admission rate among pediatrics aged 5 to 9 years decreased by 1.2% (from 9168.43 [95%CI: 9136.75–9200.10] in 2012 to 9055.06 [95%CI: 9025.16–9084.96] in 2020 per 100,000 persons). Admission rate among pediatrics aged 10 to 14 years decreased by 0.1% (from 8282.78 [95%CI: 8251.46–8314.09] in 2012 to 8270.57 [95%CI: 8241.45–8299.70] in 2020 per 100,000 persons). Admission rate among pediatrics aged 15 to 19 years increased by 0.2% (from 13,505.76 [95%CI: 13,468.63–13,542.89] in 2012 to 13,537.56 [95%CI: 13,499.57–13,575.55] in 2020 per 100,000 persons) (Fig. 2).

### 3.1. Hospital admission rates among pediatrics by age group

**Figure 3.**
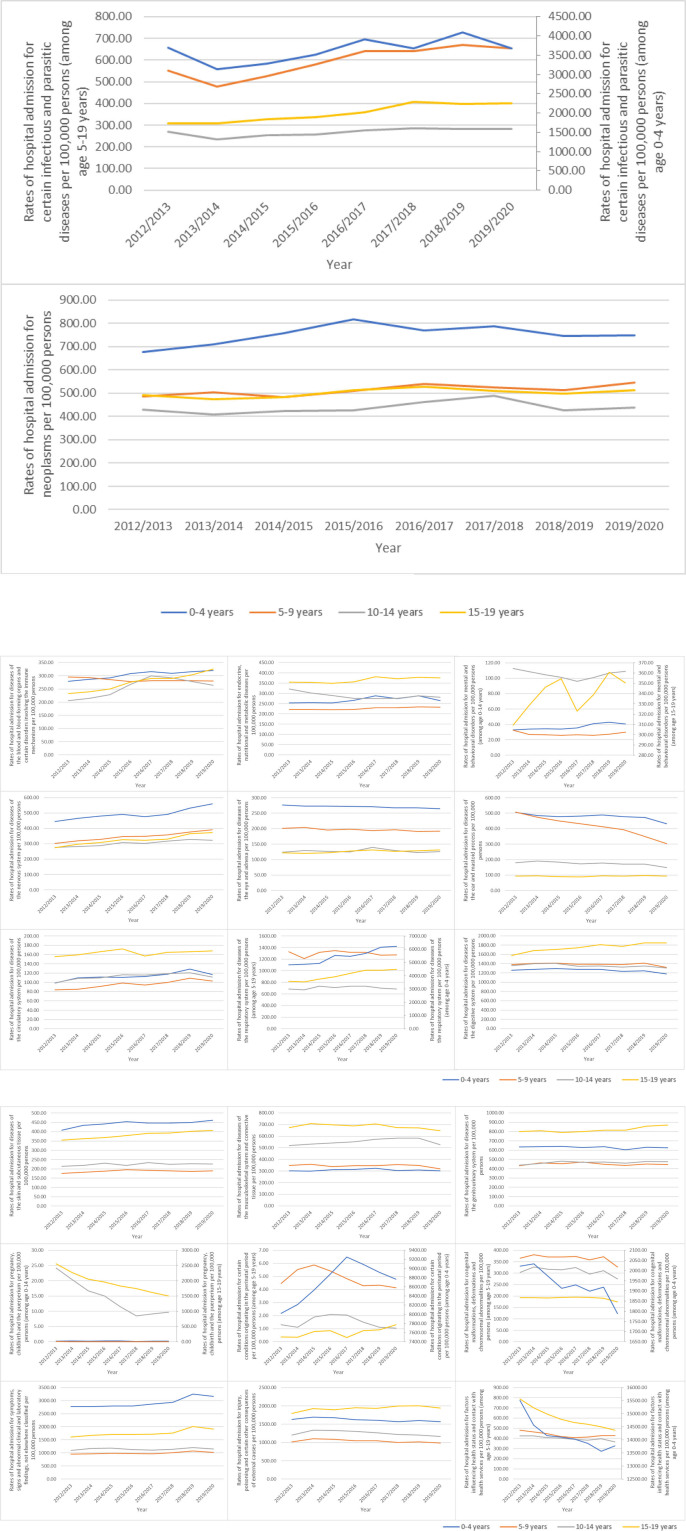
Admission rate stratified by age group.

Most pediatric admissions were more common in the 0 to 4 years age range and included the following: certain infectious and parasitic diseases, neoplasms, diseases of the blood and blood-forming organs and certain disorders involving the immune mechanism, diseases of the nervous system, diseases of the eye and adnexa, diseases of the ear and mastoid process, diseases of the respiratory system, diseases of the skin and subcutaneous tissue, certain conditions originating in the perinatal period, congenital malformations, deformations and chromosomal abnormalities, symptoms, signs and abnormal clinical and laboratory findings, and factors influencing health status and contact with health services. However, for the age group from 15 to 19 years, the following causes of admission were more prevalent: endocrine, nutritional, and metabolic diseases, mental and behavioral disorders, diseases of the circulatory system, diseases of the digestive system, diseases of the musculoskeletal system and connective tissue, diseases of the genitourinary system, pregnancy, childbirth and the puerperium, and injury, poisoning and certain other consequences of external causes (Fig. 3).

## 4. Discussion

Our study examined the trends in all-cause hospital admissions among pediatric patients in England from 2012 to 2020. The main finding was that there was a slight overall decrease in the number and rate of hospital admissions among pediatric patients over the studied period. Additionally, considerable changes were observed in the number and causes of admissions among different pediatric age groups.

The overall annual number of hospital admissions among pediatrics for different causes decreased by 0.3% representing a decrease in hospital admission rate among pediatrics of 4.0%. This was lower than the rate observed in a previous study in the USA; which found that the admission rate decreased by 26.5%.^[11]^ The difference across our findings and this study could be due to the difference in outpatient management strategies across different healthcare settings. Besides, the difference in healthcare access could be another influencing factor.

The decrease in hospital admissions in our study is likely due to a number of explanations. Firstly, improved primary care services and increased availability of services such as minor ailments clinics, where children can receive treatment without needing to be admitted to hospital. A large number of emergency department (ED) visits in the UK are for medical conditions that can be managed without professional medical intervention.^[12–16]^ Fielding et al (2015) estimated that at least 5% of ED attendances concerned such conditions,^[13]^ and Hendry et al (2005) found that 79% of the pediatric ED visits were for minor injuries and illnesses.^[17]^ Furthermore, approximately 10% of infants attending an ED in a large district general hospital had no coded diagnosis and likely attended the ED for minor illnesses and general advice only.^[18]^ National initiatives that encourage the use of alternative healthcare services such as telephone help lines, websites providing health advice, nurse-led minor illness clinics, walk-in clinics, and community pharmacy services^[14]^ could explain the slight decrease in hospital admission and hospital admission rate among pediatrics. Secondly, improved public health efforts such as vaccinations, which reduce the need for hospital admissions. Cecil et al (2018) suggested that a lack of preventive primary care, including vaccinations and development checks, is strongly linked to a greater risk of hospital admission in children.^[19]^ For example, it was found that there was a 4-fold increased risk of vaccine-preventable hospital admissions that increased across childhood. Cecil et al (2018) estimated that, in the UK, approximately 2000 preschool children are admitted to the hospital each year for problems that could have been prevented with basic vaccinations.^[19]^ However, the observed decline in admission rate could be due to changes in ICD coding practices or hospital policies; which could not be checked through the use of administrative healthcare data.

Thirdly, improved management of long-term conditions helps reduce the need for hospital admission. For instance, in England alone, there are around 1.7 million children and young people who are living with long-term conditions such as asthma, diabetes, and epilepsy. However, NHS Digital data showed that there has been a reduction in the number of children and young people with such conditions who are admitted to hospitals.^[20]^ This suggests that the NHS is managing long-term conditions among children in the community in an effective manner.

Our findings showed that the most frequent causes of hospital admission among pediatrics were factors influencing health status and contact with health services, followed by certain conditions originating in the perinatal period, and diseases of the respiratory system, accounting for 21.1%, 11.8%, and 11.2%, respectively. The increased hospital admissions in England due to factors influencing health status and contact with health services suggests that there is an increased level of awareness among parents/guardians of children to seek medical help urgently in the event of any health problem or any factor influencing children's health status.

Conditions originating in the perinatal period are ranked the second cause of hospital admissions in the present study. The outcome of neonates undergoing hospitalization in early postnatal life is associated with prenatal and postnatal morbidities.^[6,21]^ Although the UNICEF reported that the median neonatal mortality rate decreased from 36.6 (in 1990) to 17.5 per 1000 (in 2019) globally,^[22]^ factors that complicate pregnancy and result in perinatal morbidity and mortality include congenital anomalies, preterm birth, and maternal morbidity still exist. For instance, statistics show that neonates whose mothers had identified maternal pregnancy complications were 1.4 times more likely to be admitted to neonatal care.^[23]^ In line with previous scholars, respiratory disorders were also reported to be one of the most common reasons for hospital admission among children.^[21,24]^

In our study, hospital admission rate among pediatrics for diseases of the nervous system was increased by 25% while it was decreased by 46% and 25% for pregnancy, childbirth the puerperium, and diseases of the ear and mastoid process. The prevalence of neurological disorders is significantly increasing globally, with a noticed increase among neonates, largely due to the effects of prematurity and hypoxic-ischemic encephalopathy.^[25]^ Research suggests that neurological disorders account for between 2 and 12.5% of pediatric ED visits and are associated with nearly 3 times more frequent pediatric intensive care unit admissions.^[26]^ Besides, it is worth mentioning that the increase in nervous system disease admissions could be due to the development of new technologies, the improvement in diagnostic abilities of healthcare professionals, and the advancement in healthcare provision.^[27]^

On the other hand, hospital admission due to pregnancy, childbirth, and the puerperium is approximately halved in the present study. Consistently, figures from UK statistics show that the under-18 conception rate fell from 15.8 (in 2019) to 13.1 conceptions per 1000 women in 2020, continuing the downward trend of decreasing conception rates that has been seen since 2007.^[28]^ The data suggest that improved access to contraception has reduced the risk of unintended pregnancies. Furthermore, it indicates improved access to antenatal care, midwifery, and postnatal care, which has enabled better monitoring, and management of pregnancies and postpartum complications, and reduced the need for hospital admission.

The age group 0 to 4 years in our study accounted for the highest number of hospital admissions among pediatrics in the UK likely due to their greater susceptibility to illness,^[29,30]^ lower levels of immunity,^[31]^ and malnutrition.^[32]^ Moreover, their perinatal complications and immature immune systems are other influencing factors that increase the risk of admissions among this age group.^[33,34]^ Consequently, it is not surprising to notice a slight increase in the rates of hospital admission among this age group. Not only among younger ages but also the rate of hospital admission is slightly increased among 15 to 19 years, this could be explained by the role of lifestyle and the changes they experience due to the physiological and psychosocial changes associated with adolescence in the causation of chronic diseases such as diabetes mellitus and mental illnesses.^[35]^ A review of 9 cohorts and 3 cross-sectional studies among adolescents found that unhealthy diets are associated with an increased risk of mental illness.^[36]^ Furthermore, meta-analyses of prospective cohort studies concluded that physical activity is inversely associated with depression in youth and children.^[37]^ Research also has found that adolescent obesity increases the risk of depression and other mental health symptoms.^[38]^ Another explanation can be attributed to the abuse of drugs and alcohol among adolescents; UK teenagers report having some of the highest rates of alcohol use in Europe. Statistics in Scotland indicate that there has been a 200% increase in hospitalizations of 15 to 24-year-olds due to alcohol-related causes, and a 500% increase in the number of 15 to 24-year-olds treated for alcohol psychosis.^[39]^ A significant number of children and young people are still being admitted to the hospital for alcohol-related harm including intentional self-harm.^[40]^ Social aspects, developmental problems, availability of substances, and mental health should be considered among the 15 to 19 age group who attended hospitals.

The study findings highlight the importance of launching educational health campaigns targeting the community. These campaigns should increase community health literacy concerning modifiable risk factors that can decrease the burden of their associated complications that could lead to hospital admissions. Besides, decision-makers should launch community-based preventive healthcare services for high-risk populations. This includes adolescent mental health and substance use programs.

This study is limited in its ability to demonstrate any form of causality between the variables due to its ecological design and the lack of data at the individual level. Furthermore, the researcher was unable to obtain sufficient information on other associated variables such as gender and educational level. In order to address this limitation, we stratified admission rates by age groups. Despite that this approach alone does not eliminate the risk of potential residual bias, it demonstrated the variability in admission rates across age groups. Finally, the time frame for this study was restricted to be between 2012 and 2020 due to the unavailability of stratified data by age before 2012 and due to the COVID-19 pandemic, which has affected the hospitalization pattern significantly and changed the normal pattern of admission for the majority of health conditions. The use of administrative healthcare data is prone to diagnostic inaccuracy due to coding errors.

## 5. Conclusion

This study underscores critical trends in pediatric hospital admissions in England. It indicates a minor decrease in overall admissions, but significant increases in cases associated with the nervous system, blood disorders, and immune conditions, particularly among the 0 to 4 age group. The primary focus of healthcare practitioners should be on preventive care and early intervention, particularly for vulnerable young children and conditions that are rising. In order to improve pediatric health outcomes and inform more effective healthcare strategies, future research should investigate the underlying causes of these increases, the impact of socio-economic factors, and the efficacy of preventive measures. Moreover, primary care-based studies are warranted to examine the difference between primary care and secondary care settings and have more comprehensive findings from both settings.

## Acknowledgments

We would like to acknowledge Princess Nourah bint Abdulrahman University Researchers Supporting Project number (PNURSP2025R483), Princess Nourah bint Abdulrahman University, Riyadh, Saudi Arabia.

## Author contributions

**Conceptualization:** Asaleh El-Qasem, Esra' O. Taybeh, Abdallah Y. Naser.

**Data curation:** Asaleh El-Qasem, Abdallah Y. Naser.

**Formal analysis:** Abdallah Y. Naser.

**Funding acquisition:** Alaa A. Alsharif.

**Investigation:** Asaleh El-Qasem, Esra' O. Taybeh, Hassan Alwafi, Adnan Taybeh, Alaa A. Alsharif, Ahmed M. Al Rajeh, Jaber S. Alqahtani, Abdulelah M. Aldhahir, Abdullah A. Alqarni, Anan S. Jarab, Yosra J. Alhartani, Abdallah Y. Naser.

**Methodology:** Abdallah Y. Naser.

**Project administration:** Abdallah Y. Naser.

**Resources:** Asaleh El-Qasem, Esra' O. Taybeh, Hassan Alwafi, Adnan Taybeh, Alaa A. Alsharif, Ahmed M. Al Rajeh, Jaber S. Alqahtani, Abdulelah M. Aldhahir, Abdullah A. Alqarni, Anan S. Jarab, Yosra J. Alhartani, Abdallah Y. Naser.

**Software:** Abdallah Y. Naser.

**Supervision:** Esra' O. Taybeh, Abdallah Y. Naser.

**Validation:** Asaleh El-Qasem, Esra' O. Taybeh, Alaa A. Alsharif, Yosra J. Alhartani, Abdallah Y. Naser.

**Visualization:** Asaleh El-Qasem, Esra' O. Taybeh, Abdallah Y. Naser.

**Writing – original draft:** Asaleh El-Qasem, Esra' O. Taybeh, Abdallah Y. Naser.

**Writing – review & editing:** Asaleh El-Qasem, Esra' O. Taybeh, Hassan Alwafi, Adnan Taybeh, Alaa A. Alsharif, Ahmed M. Al Rajeh, Jaber S. Alqahtani, Abdulelah M. Aldhahir, Abdullah A. Alqarni, Anan S. Jarab, Yosra J. Alhartani, Abdallah Y. Naser.
